# Embryonic bone morphogenetic protein and nodal induce invasion in melanocytes and melanoma cells

**DOI:** 10.1242/bio.032656

**Published:** 2018-05-01

**Authors:** Tobias Sinnberg, Heike Niessner, Mitchell P. Levesque, Christoph Dettweiler, Claus Garbe, Christian Busch

**Affiliations:** 1 Section of Dermato-Oncology, Department of Dermatology, Tuebingen University Hospital, Liebermeisterstrasse 25, 72076 Tuebingen, Germany; 2 Department of Dermatology, UniversitaetsSpital Zuerich, Gloriastrasse 31, 8091 Zuerich, Switzerland

**Keywords:** BMP, Nodal, Melanoma, Melanocytes, Epithelial-mesenchymal transition

## Abstract

Despite recent progress in melanoma therapy via inhibition of activated oncogenes or immune stimulation, most stage IV melanoma patients still have limited survival times. Existing therapeutic approaches eventually fail to prevent further invasion and metastasis, which is driven by a morphological process termed epithelial-mesenchymal transition (EMT). We previously demonstrated that inhibition of EMT in melanoma cells via antagonizing the bone morphogenetic protein (BMP)-pathway abrogated EMT and neural crest migration of melanoma cells in chick embryos. Here, we show that BMP-2 is highly expressed in invasive melanoma cells and is elevated in the serum of stage IV melanoma patients compared to stage IB-IIC patients and healthy controls. Highly BMP-2-expressing melanoma cells display enhanced invasion in the rhombencephalon of the chick embryo. In addition to driving neural crest migration in the zebrafish embryo, the agonists BMP-2, BMP-7 and nodal induce EMT/invasion in radial growth phase melanoma cells and in human melanocytes in skin reconstructs. Blocking either BMP or nodal signaling by antagonists (noggin, lefty), or the Alk4/5/7-receptor inhibitor SB431542, decreases EMT and invasion of melanoma cells in human epidermal skin reconstructs. Together, our data suggest that inhibition of EMT-inducing pathways in melanoma might be a therapeutic approach to attenuate melanoma cell invasiveness.

## Abstract (precis)

**Summary:** We show that bone morphogenetic protein and nodal drive epithelial-mesenchymal transition (EMT) and invasiveness in melanoma cells, induce EMT and a melanoma-like invasive phenotype in melanocytes.

## INTRODUCTION

The majority of melanomas constitutively express oncogenic BRAF and/or NRAS (Raaijmakers et al., 2016), driving malignant proliferation. BRAFV600E/K in particular can be used as a therapeutic target with clinically impressive response rates (Long et al., 2015); however, results are not lasting in the majority of patients due to development of drug resistance (Poulikakos et al., 2010). The second currently applied therapeutic approach for metastatic melanoma is immune checkpoint inhibition via targeting of CTLA-4 and/or PD-1 (Drake et al., 2014), which also reaches high initial response rates (Hodi et al., 2016) followed by acquired resistance towards PD-1 antibodies in up to 25% of patients (Zaretsky et al., 2016; Shin et al., 2017). Together, both therapeutic approaches bear intrinsic drawbacks and may eventually lead to ∼40% of long-term survivors. The majority of melanoma patients treated under real-life conditions, outside of clinical trials with stringent exclusion criteria, are still progressing towards a fatal outcome (Forschner et al., 2017). In addition to uncontrolled (malignant) proliferation, the second crucial trait of malignancy is invasion and metastasis, which finally leads to a deleterious outcome. The basic morphological process underlying invasion and metastasis is epithelial-mesenchymal transition (EMT).

The transformation of a compact epithelial cell compound into loosely migrating mesenchymal cells is designated as EMT. The term EMT was originally used to describe the outgrowth of a mesenchymal-like marginal area from chick embryo lens epithelium in tissue culture (Greenburg and Hay, 1982). In the embryo, EMT occurs during mesoderm formation in the primitive streak and emigration of neural crest cells from the placodal structure of the neural crest. EMT is induced by the ligand activin/nodal of the TGF-β superfamily, among others regulated by the nodal antagonist lefty, which determines the asymmetric development of the body (Meno et al., 1996). Today, the similarity of invasion stages of cancer cells with embryonic EMT is widely acknowledged (Harper et al., 2018), and the genetic signatures of the proliferative and invasive phenotypes of melanoma cells are known (Hoek et al., 2008).

In melanoma, EMT occurs in the early phase, when transformed melanocytes break through the basal lamina of the epidermis, and later on in the phase of metastasis, when the cells enter the blood vessels and invade other organs. Melanocytes are derived from the embryonic neural crest (Le Douarin and Kalcheim, 1999). In the melanocyte lineage, EMT occurs during neural crest cell emigration from the neural tube, which is induced by autocrine secretion of TGF-β superfamily members BMP-4 and -7 (bone morphogenetic protein 4 and 7) in the neural crest compartment (Liem et al., 1995). In the adjacent neuroepithelium, EMT is inhibited by the expression of noggin, which also belongs to the TGF-β superfamily and neutralizes BMPs by dimerization (Zimmerman et al., 1996; McMahon et al., 1998; Zhu et al., 2006). In the melanocyte lineage EMT is reversible, since after neural crest migration the melanoblasts settle down in the epidermis and once again acquire an epithelial phenotype. During melanoma progression a switch of embryonic EMT-inducing transcription factors is observed (Caramel et al., 2013) and plays a crucial role in plasticity mediated therapy resistance (Richard et al., 2016).

Melanoma cells are malignantly transformed melanocytes with the respective embryonic neural crest history. In line, melanoma cells can express BMP-2, -4, and 7 (Rothhammer et al., 2005). To study the expression and regulation of EMT and cell migration in melanoma cells, we injected melanoma cells into the neural tube of a 2-day-old chick embryo and thus into the embryonic environment of the neural crest. Human SKMEL28 (Schriek et al., 2005) and mouse B16-F1 (Oppitz et al., 2007) melanoma cells spontaneously integrated into the neural crest. Together with the chick autochthonous neural crest, the melanoma cells performed EMT and resumed neural crest migration along the medial and lateral pathways.

The role of BMP-2 and its antagonist noggin in neural crest migration of transplanted cells was first established in neural stem cells from the subventricular zone of the mouse forebrain. *In vitro* the neural stem cells form neurospheres. BMP-2 treatment of the neurospheres induces EMT and a neural crest phenotype *in vitro* (Sailer et al., 2005). Neurospheres transplanted into the neural tube of chick embryos only performed neural crest migration after pre-treatment with BMP-2 (Busch et al., 2006). We therefore reasoned that neural crest migration and malignant invasion of melanoma cells could also be BMP-2-dependent. In addition to BMP-2, melanoma cells constitutively express the TGFbeta-family member nodal (Topczewska et al., 2006). We therefore included the agonist nodal, its inhibitor lefty, and the Alk4/5/7-receptor antagonist SB431542 (Laping et al., 2002) into the present study.

In the current study we observed a high BMP-2 expression in melanoma cells with an invasive phenotype. Therefore we measured the BMP-2 concentration in serum samples of controls and melanoma patients and analyzed the role of BMP and nodal for physiological neural crest migration in the zebrafish embryo. We further assessed their impact on melanoma cell proliferation and invasion in monolayer culture and organotypic skin reconstructs. Vice versa, we analyzed the effects of BMP and nodal on melanocyte proliferation and invasion.

## RESULTS

### BMP-2 is specifically up-regulated in invasive melanoma cells

**Fig. 1.**
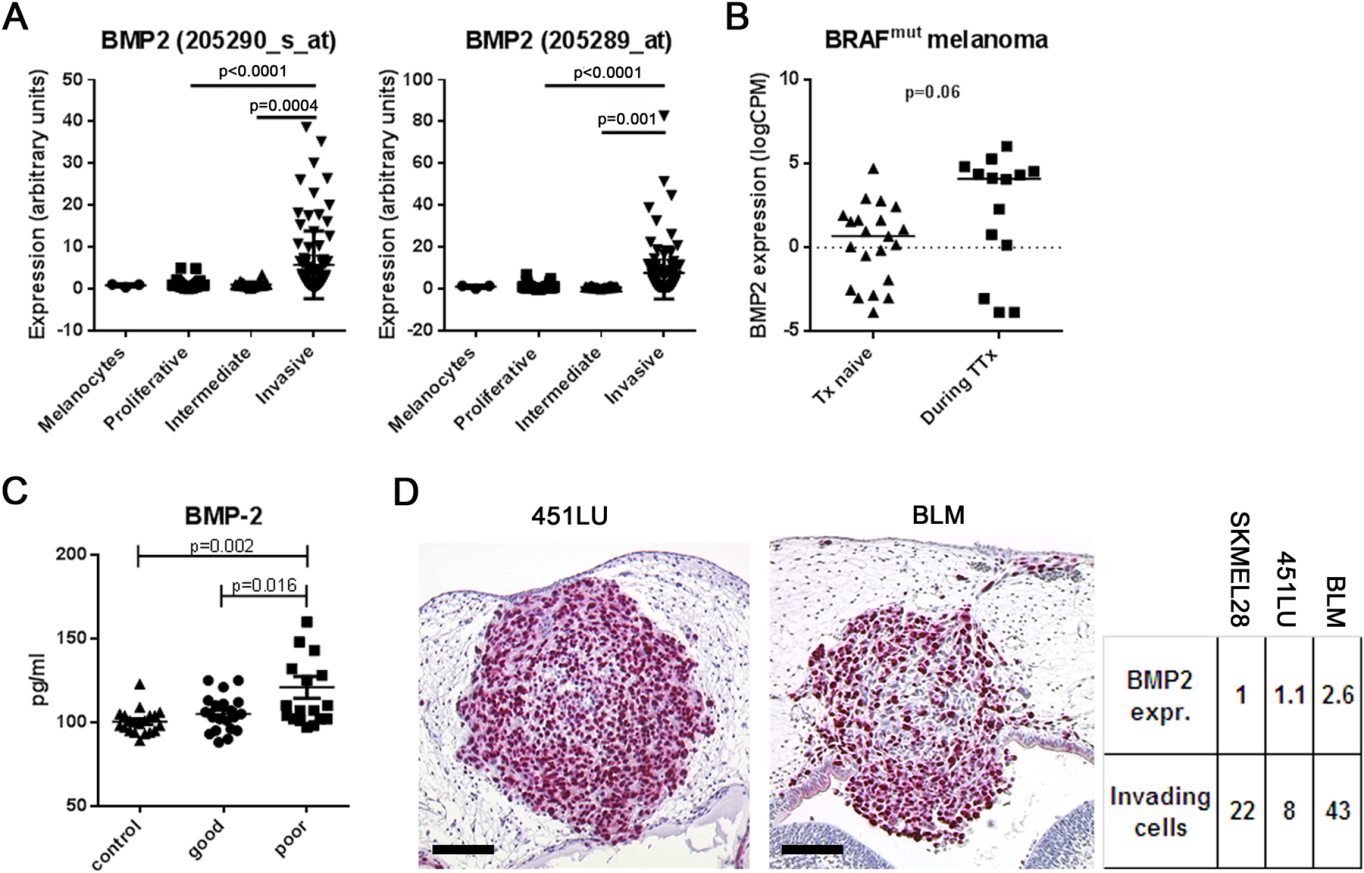
**BMP-2 is up-regulated in melanoma cells with an invasive phenotype.** (A) A melanoma database (http://www.jurmo.ch/php/genehunter.html) was screened for the expression level of BMP-2. In the four different datasets comprising melanocytes (*n*=3), proliferative (*n*=101), intermediate (*n*=26), and invasive (*n*=90) melanoma cell lines, BMP-2 was significantly up-regulated in melanoma cells with an invasive phenotype compared to cells with a proliferative phenotype (one-way ANOVA). (B) RNA-expression of BMP-2 in BRAF mutated patient-derived melanoma cells therapy (Tx)-naïve or during targeted therapy (TTx). A subset of melanoma cells from patients under TTx had a strong expression of BMP-2 compared to the Tx-naïve cohort (median 0.48 vs 4.10 counts per million, *P*=0.06, *t*-test). (C) BMP-2 serum levels in healthy controls (*n*=20) and 38 melanoma patients [*n*=20 stages IB-IIC (long survival), and *n*=18 stage IV (short survival)]. In control sera, median BMP-2 concentration was 100.5 pg/ml (89-123 pg/ml). In sera from patients with long survival it was 105.1 pg/ml (88-125 pg/ml). In sera from patients with short survival it was 120.9 pg/ml (97-201 pg/ml). The differences between controls and short-term survivors and between long-term and short-term survivors were significant (one-way ANOVA). (D) Chick embryo brain metastasis model. Histological slides were analyzed for single melanoma cell invasion in the rhombencephalon of the chick embryo and correlated with BMP-2 RNA-expression of the corresponding melanoma cell line. Expression of BMP-2 was 2.6-fold higher in highly invasive BLM compared to less invasive 451LU and SKMEL28 (SKMEL28: 22±11 invading cells per histological section, *n*=5 embryos analyzed; 451LU: 8±2, *n*=6; BLM: 43±10, *n*=8; *P*<0.05, one-way ANOVA). Scale bars: 100 µm.

The invasive potential of melanoma cells is defined by a specific gene expression pattern and thereby clearly distinguished from melanoma cells with a proliferative phenotype (Hoek et al., 2006). We analyzed the expression of BMP-2 and nodal in large numbers of melanoma cell lines attributed to either the proliferative or the invasive phenotype using a melanoma database (http://www.jurmo.ch/hopp/hopp_mpse.php). While no difference could be found between proliferative and invasive melanoma cells for nodal expression (not shown), the four different datasets comprising a total of 101 proliferative, 90 invasive and 26 intermediate melanoma cell gene profiles yielded a significant up-regulation of BMP-2 in all four datasets in melanoma cells with the invasive phenotype compared to cells with the proliferative phenotype (Fig. 1A). This demonstrates that BMP-2 up-regulation is a general phenomenon in invasive melanoma cells.

### MAPK inhibition is accompanied by increased expression of BMP-2 in patient-derived metastatic melanoma cells

BMP-2 is up-regulated in invasive melanoma cells and is a known inducer of EMT. BRAF activation or inhibition drives a rapid and reversible switch in EMT-inducing transcription factors (Caramel et al., 2013). In line, EMT in general is able to mediate melanoma cell plasticity and resistance to MAPK inhibitors (Richard et al., 2016). Therefore, we analyzed the RNA-expression of BMP-2 in a panel of BRAF mutated patient-derived melanoma cells (therapy-naïve and during targeted therapy using BRAF and/or MEK inhibitors) from the publically available Zurich biobank database (http://tcgabrowser.ethz.ch:3839/MCE/). Our RNAseq analysis revealed that a subset of melanoma cells from patients under MAPK-inhibition had a strong expression of BMP-2 when compared to therapy-naïve patients (median 0.48 vs 4.10 counts per million, *P*=0.06, *t*-test, Fig. 1B).

### Elevated BMP-2 serum levels correlate with short survival of stage IV melanoma patients

Due to the importance of BMP-2 on melanoma cell EMT/invasiveness described above, we tested whether BMP-2 serum levels differed between healthy controls and melanoma patients, as has been previously reported on non-small-cell lung and gastric cancer patients (Choi et al., 2012; Park et al., 2008). To this end, serum was collected from 20 healthy volunteers. In addition, melanoma patient serum samples (from the serum bank of the Department of Dermatology, University of Tuebingen, Germany) were retrieved. Two patient cohorts were chosen: 1) Melanoma patients with a negative sentinel lymph node biopsy and a long follow-up time after blood withdrawal (clinical stage IB-IIC; samples were taken before the sentinel node biopsy; *n*=20; median follow-up time since blood withdrawal: 28.7 months, 17-40 months); and 2) Stage IV melanoma patients with a short survival time after blood withdrawal (*n*=18; median time until death: 6.1 months, 2-11 months). BMP-2 serum levels were determined by ELISA. The median BMP-2 concentration in the control serum samples was 100.5 pg/ml (89-123 pg/ml). The median BMP-2 concentration in the sera from stage IB-IIC patients was 105.1 pg/ml (88-125 pg/ml) and in the sera from stage IV patients it was 120.9 pg/ml (97-201 pg/ml) (Fig. 1C). The difference between control sera and stage IB-IIC sera was not significant (*P*=0.67, one-way ANOVA); however, the differences between control sera and stage IV sera, and between stage IB-IIC sera and stage IV sera were significant (*P*=0.002 and *P*=0.016, respectively, one-way ANOVA). To compare the serum BMP-2 levels to established clinical melanoma serum prognosis markers as internal quality control, lactate dehydrogenase (LDH) and S100 protein levels were also determined in the same serum samples. As expected, LDH and S100 values were normal in the sera from stage IB-IIC patients (mean LDH: 200.1 U/l; cutoff: <250 U/l; mean S100: 0.058 µg/l; cutoff: <0.1 µg/l), while stage IV patients had elevated LDH and S100 values (281.8 U/l and 0.503 µg/l, respectively), verifying the intact quality of the sera used for analysis. Together, an increased serum BMP-2 level correlates with short survival in stage IV melanoma patients. This novel finding in melanoma patients confirms what has previously been reported for non-small-cell lung and gastric cancer patients.

### BMP-2 RNA expression is associated with enhanced invasiveness of melanoma cells in the chick embryo

For the following experiment we used pre-existing histological slides of our chick embryo model (Busch et al., 2012; Sinnberg et al., 2018) and correlated single melanoma cell invasion in the rhombencephalon of the chick embryo with BMP-2 RNA-expression of the corresponding melanoma cell line. The expression of BMP-2 was 2.6-fold higher in BLM compared to 451LU and SKMEL28 (Fig. 1D). We observed that the high-BMP-2-expressing metastatic cell line BLM had an increased invasive capacity when compared to the lower-BMP-2-expressing metastatic cell lines 451LU and SKMEL28 (SKMEL28: 22±11 invading cells per histological section, *n*=5 embryos analyzed; 451LU: 8±2 invading cells per histological section, *n*=6 embryos analyzed; BLM: 43±10 invading cells per histological section, *n*=8 embryos analyzed; *P*<0.05, one-way ANOVA).

### In zebrafish embryos, blocking of BMP signaling abrogates neural crest cell migration

**Fig. 2.**
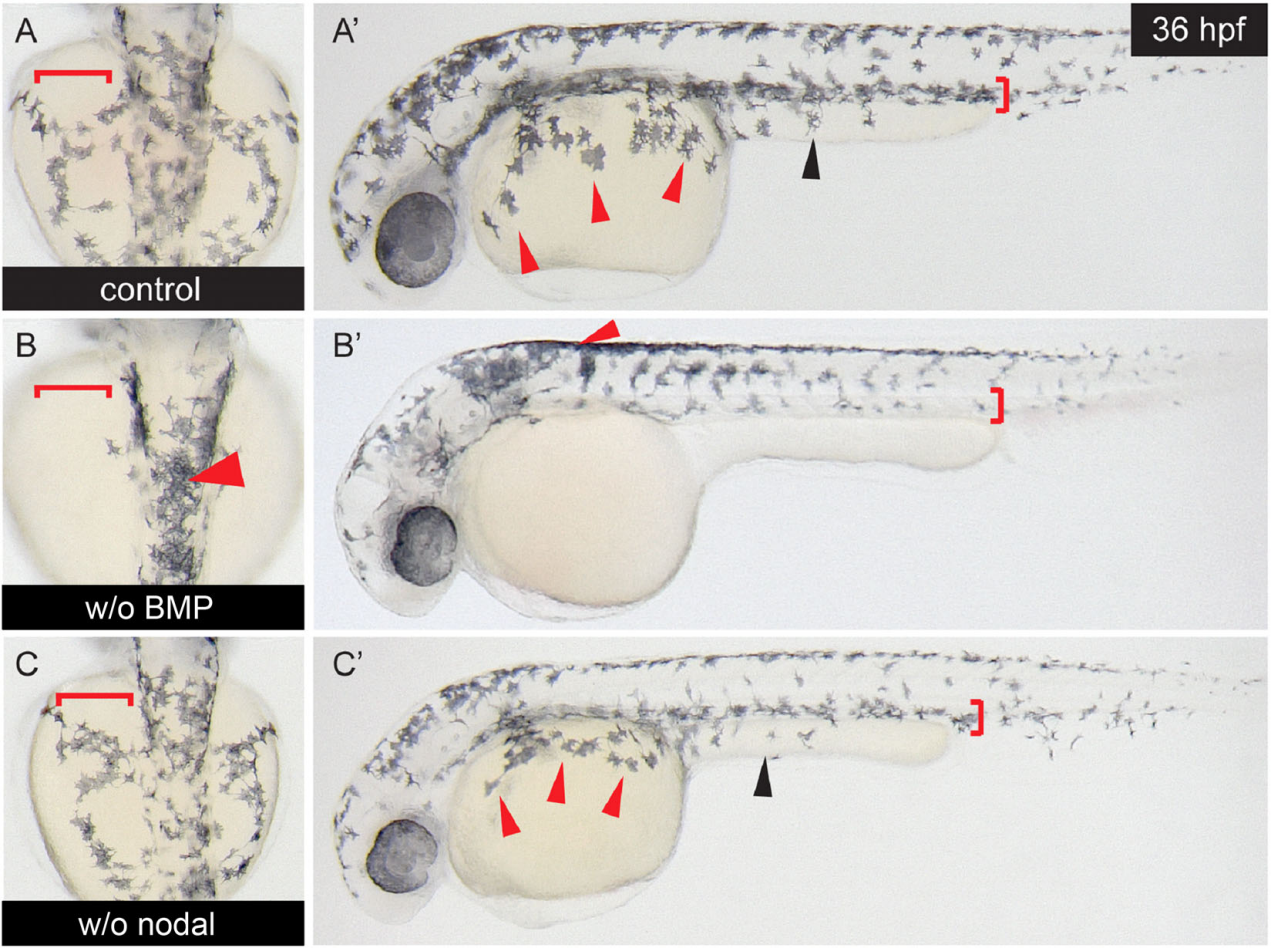
**Blocking of BMP signaling, but not of nodal signaling, inhibits autochthonous neural crest migration in zebrafish embryos.** Zebrafish embryos (*n*=6 per group) were exposed to 10 µM of the BMP type I receptor-antagonist dorsomorphin or to 30 µM of the Alk4/5/7 nodal receptor antagonist SB431542 6 hpf. Embryos at 36 hpf were analyzed for neural crest cell migration. (A,A′) Control embryos show a normal pattern of neural crest cell migration along the dorsolateral and the dorsoventral migration pathways in upper and lateral view. (B,B′) Upon dorsomorphin treatment neural crest migration is impaired along both pathways. (C,C′) SB431542 does not influence neural crest migration in zebrafish embryos. Arrowheads indicate melanocytes that are supposed to populate the yalk sac (red arrowheads) or the yalk extension (black arrowheads). The areas into which the melanocytes migrate by the dorso-ventral or dorso-lateral pathways are marked by red brackets.

To analyze a possible effect of BMP or nodal on cell migration, we first tested whether the BMP or nodal signaling pathways were involved in physiological neural crest cell migration in zebrafish embryos. It is already known for numerous other species that BMPs induce the neural crest (e.g. LaBonne and Bronner-Fraser, 1998; Nguyen et al., 1998; Kanzler et al., 2000). We used dorsomorphin to block BMP signaling and the ALK4/5/7 nodal/activin receptor antagonist SB431542 to block nodal signaling. Dorsomorphin selectively inhibits the BMP type I receptors ALK2, ALK3 and ALK6 and thus blocks BMP-mediated SMAD1/5/8 phosphorylation (Yu et al., 2008). As a model organism, we chose the zebrafish embryo. Embryos were treated with 10 µM dorsomorphin or 30 µM SB431542 at 6-8 h post fertilization (hpf) and photographed after 36 hpf. In control embryos, neural crest cells performing dorsoventral and dorsolateral migration were observed (Fig. 2A). Dorsomorphin-treatment abrogated dorsoventral and dorsolateral neural crest cell migration (Fig. 2B). Treatment with SB431542 had no effect on neural crest cell migration in zebrafish embryos (Fig. 2C). Together, these data show and confirm that the physiological neural crest induction (Nguyen et al., 1998) and migration in the zebrafish embryo depends on endogenous BMP signaling.

### Agonists (BMP-2, nodal) and antagonists (noggin, lefty, SB431542) have no influence on cell cycle, colony formation or chemosensitivity of melanoma cells

**Fig. 3.**
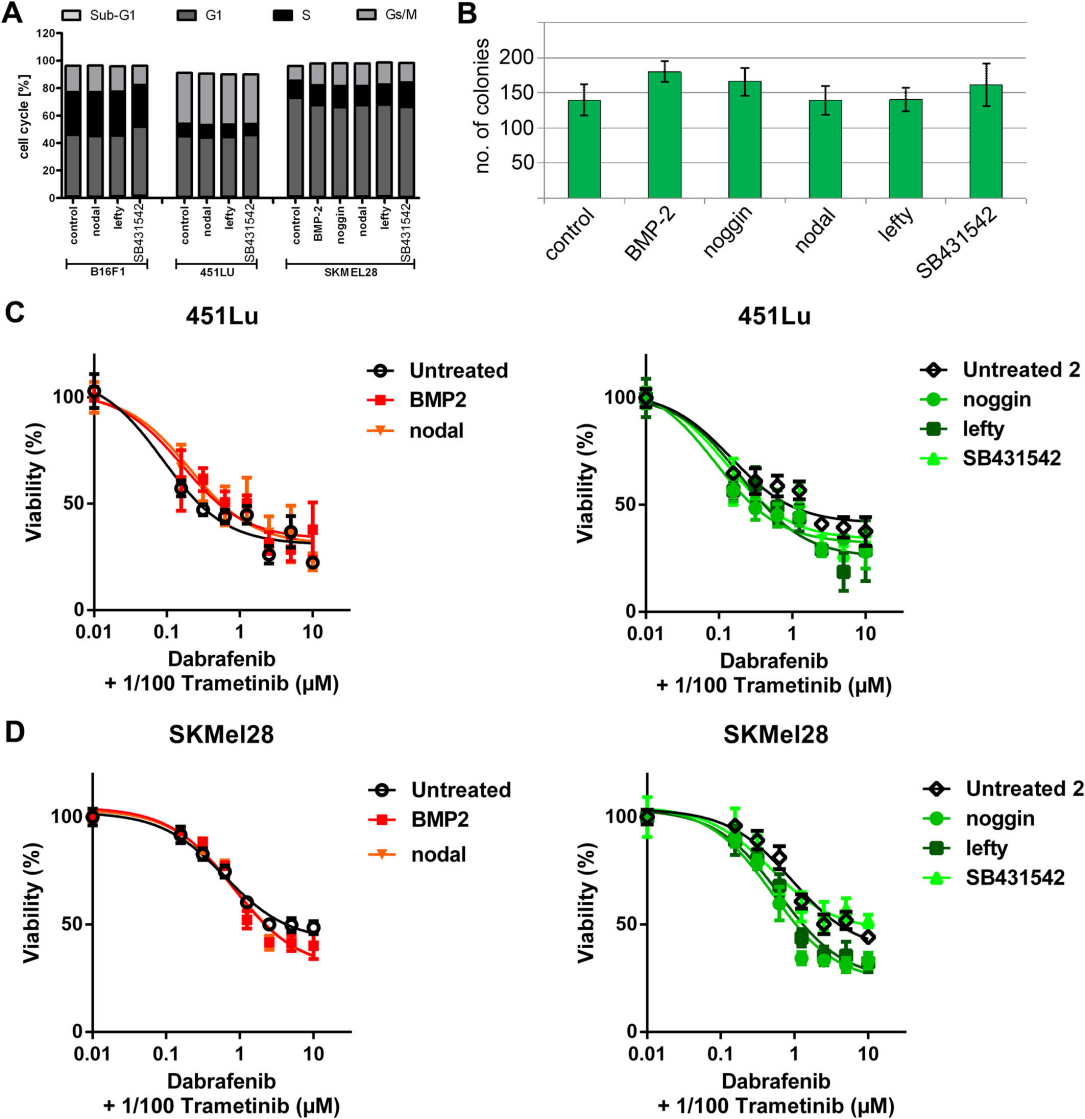
**Morphogens have no influence on cell cycle, colony formation or chemosensitivity in melanoma cells.** (A) FACS cell cycle analyses (PI-staining) of B16F1, 451LU and SKMEL28 melanoma cells were conducted after treatment with agonists or antagonists. No changes in cell cycle distribution were detected. (B) No significant influence on colony formation (in soft agar) of agonists and antagonists was observed in SKMEL28 cells (one-way ANOVA). (C,D) Chemosensitivity/proliferation assays (MUH) were performed 72 h after treatment of SKMEL28 and 451LU melanoma cells with dabrafenib and trametinib in combination with the morphogens. No significant influence of agonists or antagonists was detected.

To assess the possible influence of the agonists BMP-2 and nodal, their antagonists noggin and lefty and the nodal receptor antagonist SB431542 on cell proliferation, we conducted cell cycle analyses of B16F1, 451LU and SKMEL28 melanoma cells after treatment with agonists or antagonists. No changes in cell cycle distribution could be detected (Fig. 3A). Likewise, no influence on colony formation (in soft agar) of agonists and antagonists was observed in SKMEL28 cells (Fig. 3B). Further, we performed chemosensitivity arrays 72 h after treatment of SKMEL28 and 451LU melanoma cells (both carrying a BRAF mutation) with the BRAF-antagonist dabrafenib in combination with the MEK-inhibitor trametinib in combination with agonists and antagonists. Here too, no influence of agonists and their antagonists was detected (Fig. 3C,D). Together, these data show that BMP-2, nodal and their respective antagonists neither influence melanoma cell proliferation nor sensitize melanoma cells for the current standard treatment regimen for BRAF-mutated metastatic melanoma patients (BRAF±MEK-inhibition).

### Antagonists decrease while agonists induce melanoma cell invasion in human epidermal skin reconstructs; agonists switch radial growth phase SBCL2 melanoma cells to an invasive phenotype

**Fig. 4.**
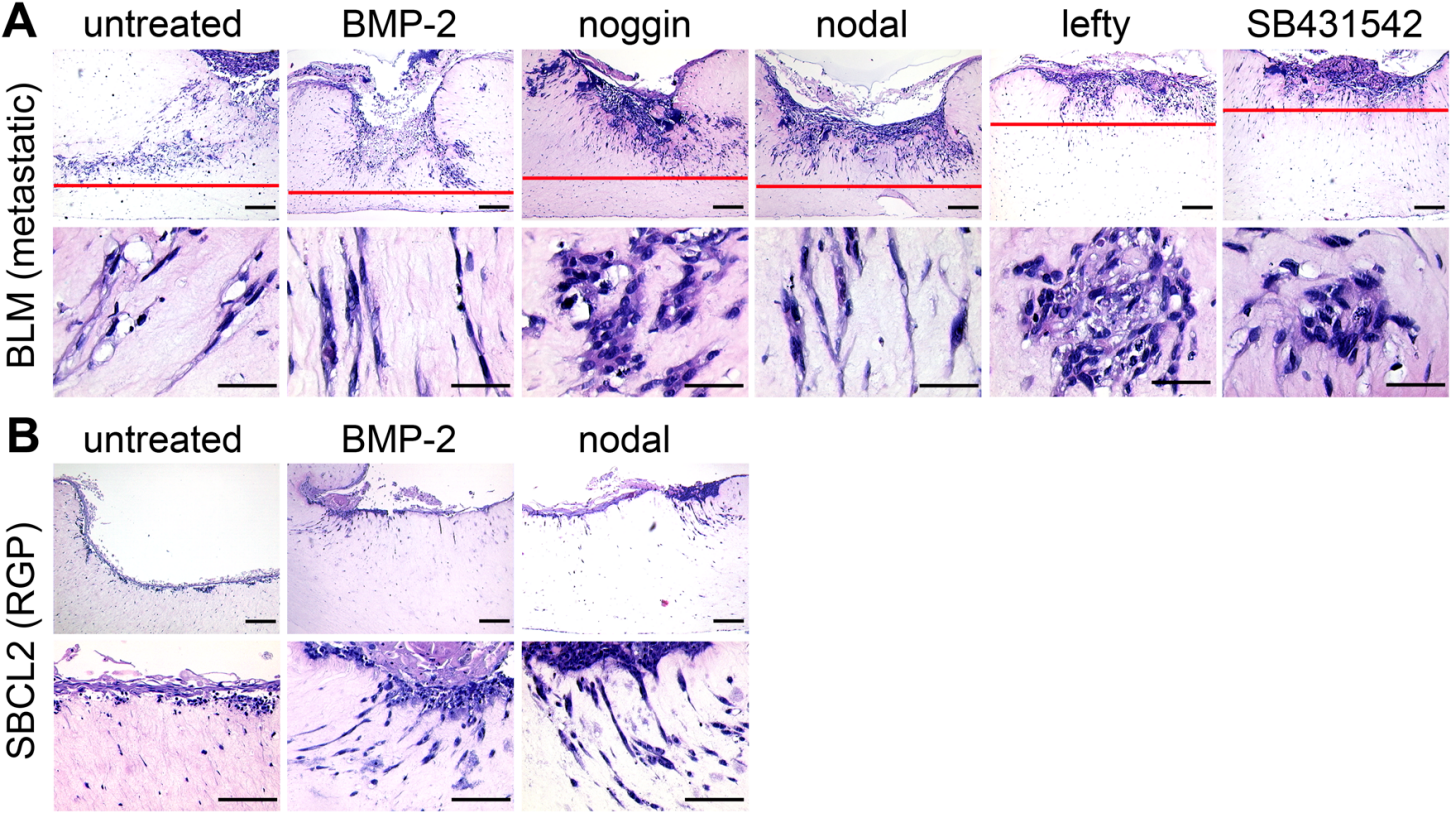
**BMP and nodal induce invasion of metastatic and radial growth phase melanoma cells in human epidermal skin reconstructs.** Control and pre-treated BLM (metastatic) or SBCL2 (radial growth phase) melanoma cell aggregates were seeded onto human epidermal skin reconstructs (*n*=3 per treatment group). After 16 days of further reconstruct formation, invasion of melanoma cells was assessed by histology. (A) BMP-2 and nodal increased the destruction of the epidermal part and invasion into the dermal part of the reconstructs by BLM cells; noggin, lefty and SB431542 had opposing effects. Agonist-treated melanoma cells depict a stretched, mesenchymal morphology; antagonist-treated melanoma cells showed a compact, epithelial morphology in the upper dermal part (lower row). Scale bars: 200 µm (upper row) and 50 µm (lower row). (B) In untreated SBCL2 cells, no invasion was found (left column). BMP-2 and nodal induced invasion and a mesenchymal, stretched morphology in SBCL2 cells (middle and right columns). Scale bars: 200 µm (upper row) and 50 µm (lower row).

To test the efficacy of the morphogens to alter invasive melanoma growth in a more sophisticated environment, pre-treated BLM melanoma cell aggregates were seeded onto human epidermal skin reconstructs. After 16 days of further reconstruct formation, invasion of BLM cells was assessed by histology. Skin reconstructs with untreated, BMP-2- and nodal pre-treated melanoma aggregates depicted similar images: proliferating, massively invasive, growing primary tumors completely penetrating the entire extension of the skin reconstructs (Fig. 4A). This was even more pronounced in the BMP-2- and nodal-treated groups. However, the reconstructs with noggin-, lefty-, and SB431542-pre-treated BLM aggregates showed only superficial primary tumors with considerably less destruction and invasion. Interestingly, in the control, BMP-2 and nodal groups, the invading melanoma cells depicted a mesenchymal, stretched morphology, while in the antagonist groups, clusters of epithelial-like, rounded, aggregated BLM cells prevailed in the dermal part of the skin reconstructs (Fig. 4A, lower panel). In a second approach, we asked whether BMP-2 or nodal might be involved in the transition of radial growth phase (RGP) melanoma cells to a vertical/invasive growth phase (VGP) (Miller and Mihm, 2006). To this end, we seeded untreated, BMP-2- and nodal treated SBCL2 cells (RGP) on top of the human skin reconstructs. After 16 days, as expected (Krochmann et al., 2012), histological analysis indicated that untreated control cells proliferated in the epidermal part of the reconstruct without invasion of the dermal part (Fig. 4B). However, BMP-2- and nodal-treated SBCL2 cells invaded the dermal part of the reconstructs and depicted a mesenchymal morphology resembling the invading metastatic BLM cells. The different invasion capacities and cell morphologies 16 days after treatment of BLM melanoma cells and the induction of invasive growth in the RGP SBCL2 cells after BMP-2 and nodal treatment confirm a temporary inhibitory influence of the antagonists and an enhancing impact of the agonists on invasion in this *in vitro* skin model. Together, these results demonstrate that the agonists enhance the invasion of melanoma cells and promote the transition of RGP melanoma cells to VGP melanoma cells. In line, the antagonists inhibit invasion of melanoma cells in the skin reconstructs. These findings confirm and extend our previously reported data of inhibition of neural crest cell-like migration of melanoma cells in the chick embryo by the BMP-antagonist noggin (Busch et al., 2007).

### Agonists BMP-2, BMP-7 and nodal do not influence proliferation, but induce invasion in melanocytes *in vitro* and in human epidermal skin reconstructs

**Fig. 5.**
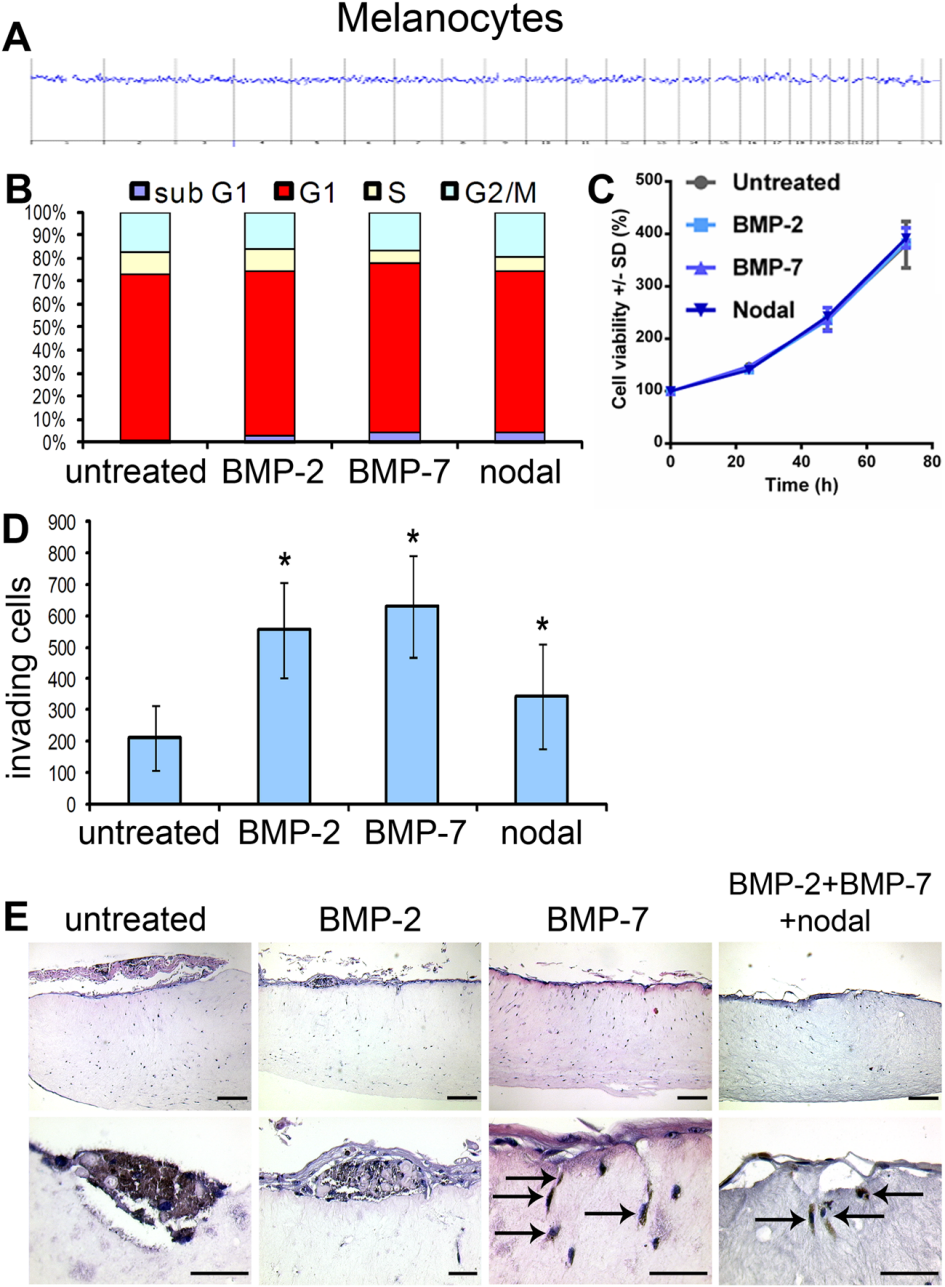
**BMP-2, BMP-7 and nodal have no influence on cell cycle, but promote invasion in primary human melanocytes.** (A) Comparative genomic hybridization (CGH) of HEM1 melanocytes. No chromosomal aberrations were detected. (B) FACS cell cycle analyses (PI-staining) of HEM1 melanocytes were conducted after treatment with the agonists. No significant changes in cell cycle distribution were detected. (C) Proliferation assays (MUH) were performed 24 h after pre-treatment of HEM1 melanocytes with the agonists. No influence on proliferation was detected. (D) HEM1 melanocytes pre-treated with BMP-2, BMP-7 or nodal for 24 h were assessed for invasion in a Matrigel-coated Boyden chamber assay (*n*=4 per group). All three agonists increased the number of invasive melanocytes compared to the control cells (*P*<0.05, one-way ANOVA). (E) The influence of the agonists was assessed on induction of invasion of HEM1 melanocytes in skin reconstructs (*n*=3 per group). Control and BMP-2 pre-treated melanocytes were confined to the epidermal part of the reconstructs. Melanocytes pre-treated with BMP-7 or all three agonists could be detected in the dermal part of the reconstruct with a stretched, mesenchymal-like morphology and typical pigmentation. Scale bars: 200 µm (upper row) and 50 µm (lower row).

To compare the malignantly transformed melanoma cells to non-transformed melanocytic cells, we conducted a similar set of experiments using human foreskin epidermal melanocytes. This experimental approach was crucial to determine whether BMP or nodal signaling was sufficient to induce ‘malignant’ characteristics (e.g. enhanced proliferation or invasion) in benign cells without genomic aberrations or activated oncogenes. To exclude possible genomic alterations, we first performed a comparative genomic hybridization (CGH) of the HEM1 melanocytes (Fig. 5A). The CGH proved the benign nature of the melanocytes. To analyze a possible influence of the agonists BMP-2, BMP-7 and nodal on proliferation of the HEM1 melanocytes, we performed cell cycle analyses, showing that the pre-treatment with the agonists caused no changes in the cell cycle distribution after 24 h (Fig. 5B). In line, we detected no differences in cellular proliferation upon stimulation of the melanocytes with either BMP-2, BMP-7, or nodal after 24 h (Fig. 5C). To screen for possible induction of invasion by the agonists, we performed a Boyden chamber invasion assay. HEM1 melanocytes were pre-treated with the agonists in monolayer cell culture in six-well plates for 24 h prior to conducting the invasion assay. After 24 h, melanocytes that had penetrated the Matrigel-coated membrane were stained and counted. BMP-2, BMP-7 and nodal significantly increased the number of invasive HEM1 melanocytes two- to threefold (Fig. 5D). Together, these data show that BMP-2, BMP-7 and nodal have no influence on the cell cycle in primary human melanocytes. Further, these results demonstrate an induction of migration and invasion with reduced adhesion properties in melanocytes by all three agonists.

To finish, we tested the efficacy of the agonists to induce invasion in HEM1 melanocytes in human epidermal skin reconstructs. Pre-treated (24 h) melanocytes were seeded on top of epidermal skin reconstructs. After 16 days of skin reconstruct formation, invasion was assessed by histology. In skin reconstructs with untreated HEM1 melanocytes we found single melanocytes and groups of melanocytes in the epidermal compartment of the reconstruct among keratinocytes (Fig. 5E). In the BMP-2 pre-treated melanocytes a similar result was obtained. However, melanocytes pre-treated with BMP-7 or with BMP-2+BMP-7+nodal yielded similar results in the epidermal compartments, while, in addition, single pigmented, stretched melanocytes could well be distinguished invading the upper level of the dermal part of the reconstructs (Fig. 5E, arrows). The induction of invasive migration in melanocytes in human skin reconstructs by BMP and/or nodal corroborates with our previous observation of induction of a similar invasive migration of human melanocytes in the optic cup of the chick embryo (Busch et al., 2013).

## DISCUSSION

In the current study, we showed the importance of BMP signaling for neural crest migration/EMT in the zebrafish embryo and demonstrated BMP- and nodal-dependent EMT/invasiveness of human melanoma cells and melanocytes.

EMT represents a complex change in cell morphology and migratory potential of embryonic cells and is induced in the embryo mainly by dorsalizing BMP and Wnt, and is inhibited by ventralizing sonic hedgehog and noggin signaling (Hay, 2005; Garcia-Castro et al., 2002). In particular, BMP is an inductor of neural crest cell migration, whereas noggin acts as an inhibitor (Hogan, 1996; Sela-Donenfeld and Kalcheim, 1999). EMT in the neural crest comprises two consecutive steps (Newgreen and Minichiello, 1995): first, the neural crest compartment is induced in the epithelium of the neural tube. This step is characterized by the disintegration of the basal lamina in the region of the lateral roof plate. Second, neural crest cells are induced to start migration from the dorsal edges of the neural tube along their pathways. Functionally, we asked if BMP or nodal were important for physiological neural crest cell migration in the zebrafish embryo, as described for BMP for chick (Liem et al., 1995) and *Xenopus* embryos (Fainsod et al., 1994). Blocking BMP signaling with dorsomorphin completely abrogated neural crest cell migration, in line with chick and *Xenopus* embryos. Inhibition of nodal signaling, however, had no effect on neural crest cell migration in zebrafish embryos.

Treatment with the agonists (BMPs, nodal) or the antagonists (noggin, lefty, SB431542) had no effect on melanoma cell proliferation or cell cycle and did not sensitize melanoma cells for modern pharmacological agents (dabrafenib, trametinib), indicating that the effects of BMP and nodal were independent of oncogenes driving uncontrolled proliferation. In melanoma cells, noggin, lefty and the receptor blocker SB431542 reversed the migratory mesenchymal morphological phenotype back to the aggregate epithelial morphological phenotype and ablated the capability of invasive behavior in physiological skin reconstructs. Vice versa, BMP and nodal induced invasion of the radial growth phase SBCL2 melanoma cells in the same setup. Treatment of non-transformed human melanocytes with BMPs or nodal had no impact on proliferation either, but induced a mesenchymal phenotype and invasiveness *in vitro* and in skin reconstructs, suggesting that BMP and nodal are ‘driving’ events in melanocytic cell spreading and possible key factors for melanoma progression. However, we acknowledge the preliminary nature of our study due to the limited number of cell lines used for the experimental part of this report. Further studies are needed in this important field of research to substantiate the preliminary conclusions drawn. A supporting corresponding observation (induction of invasiveness in melanocytes) was described for embryonic Notch1 (Pinnix et al., 2009).

Our current findings corroborate our previous results showing that spontaneous neural crest migration (Busch et al., 2007) and malignant invasion (Busch et al., 2008) of melanoma cells were abrogated by pre-treatment of the melanoma cell aggregates with noggin before transplantation into the chick embryo (Busch et al., 2013). Thus, the source of the BMP, which drives neural crest migration, is the melanoma cells themselves. This is in line with constitutive expression of BMPs in human melanoma cell lines and primary melanomas (Rothhammer et al., 2005) and increased BMP-7 expression in melanomas and their metastases, when compared to nevi (Rothhammer et al., 2007). The importance of BMP for melanoma cell invasion was further supported by the observation that BMP-2 induced invasion of non-transformed human melanocytes in the optic cup as ectopic site of the chick embryo (Busch et al., 2013). Using antagonists (noggin, lefty) or the receptor blocker SB431542, the described EMT switch is pharmacologically accessible via the extracellular autocrine loop of the TGF-β transduction chain (Arai et al., 2016). In this respect, the induction of the migratory/EMT phenotype has an epigenetic character, although in melanoma it meets a constitutively open BMP and nodal synthesis with the respective receptor expression. The latter findings are supported by recent data showing that the histone H3 lysine 27 (H3K27) demethylase JMJD3 epigenetically induces the BMP signaling pathway, thus promoting melanoma progression and metastasis (Park et al., 2016). In that report, BMP-4 regulated JMJD3 expression via a positive feedback mechanism. Together, these data suggest that melanoma cells constitutively express two separate EMT-inducing genes (BMP and nodal) that are both involved in EMT and invasion. This feature distinguishes melanoma cells from their embryonic precursors, the neural crest cells. Interestingly, the BMP-expression as prerequisite for melanoma invasion corresponded to the increased expression of BMP-2 in a large number of invasive melanoma cell lines compared to proliferative cell lines (Hoek et al., 2006), which was further corroborated by elevated BMP-2 serum levels, which clinically correlated with short survival times in stage IV melanoma patients. A recent report showed that melanoma brain metastases showed a progression pattern characterized by a high propensity to disseminate in patients treated with BRAF-inhibitors (Haueis et al., 2017). The authors of the latter publication speculated that this might reflect an *in vivo* manifestation of phenotype switching (EMT) in response to targeted therapy, with a predominance of the invasive/migratory tumor cell phenotype. This is further supported by the observation that expression of the EMT inducing transcription factor ZEB1 is increased in MAPK-inhibition surviving melanoma cells (Richard et al., 2016). Most recently, it was shown that activation of BMP signaling via the ligand GDF6 governs an embryonic cell gene signature to promote melanoma progression (Venkatesan et al., 2018). This suggests that blocking EMT-inducing pathways to abrogate invasion and/or metastasis might be an efficacious approach to complement modern melanoma therapies directed against cell proliferation (BRAF- and/or MEK-inhibition) or stimulation of the immune system by checkpoint inhibitor blockade. Recent clinical trials testing the compound galunisertib or LY2157299 (a small molecule inhibitor of TGF-beta RI serine/threonine kinase) that specifically block EMT-induction via inhibition of TGF-β signaling in patients with solid cancers showed clinical efficacy and an acceptable tolerability and safety profile (Rodon et al., 2015; Fujiwara et al., 2015). Likewise, GC1008 (a human anti-TGF-β monoclonal antibody that neutralizes all isoforms of TGF-β) had an acceptable safety profile and showed preliminary evidence of antitumor activity in pre-treated stage IV melanoma patients (Morris et al., 2014).

In conclusion, our data show that BMP and nodal drive EMT and invasiveness in melanoma cells, induce EMT and a melanoma-like invasive phenotype in melanocytes. These findings render BMP and/or nodal crucial pharmacological targets for the inhibition of EMT and metastasis in melanoma patients.

## MATERIALS AND METHODS

### Cell lines, primary cells, and culture

The use of human tissues was approved by the medical ethical committee of the University of Tuebingen (Tuebingen, Germany) and was performed in accordance with the Declaration of Helsinki Principles. Every patient signed a consent form prior to surgery, allowing the generation of melanoma tissue bank, and the use of such tissue for cell isolation for individual chemosensitivity testing and research purposes.

The following melanoma cell lines were used in this study: human SKMEL28, BLM, 451LU, and SBCL2, and murine B16F1. Melanoma cells were cultured in RPMI 1640 medium supplemented with 10% fetal bovine serum (FBS), 1% penicillin, and streptomycin, 1% L-Glutamine. Media was changed at 48 h intervals, and cells were passaged upon confluence. All cell cultures were maintained at 37°C in a 95% air / 5% CO_2_ atmosphere at 100% humidity. Human primary melanocytes HEM1 were isolated from neonatal human foreskin samples as described (Mancianti et al., 1988) and cultivated in Dermalife M medium (Cell Systems, Troisdorf, Germany). Melanocytes were expanded until confluency.

### Comparative genomic hybridization (CGH)

CGH of human melanocytes was performed as described elsewhere (Held et al., 2013).

### Treatment of cells with pharmacological agents and morphogens

The pharmacologic agents dabrafenib and trametinib (both from Hycultec, Beutelsbach, Germany), alone or in combination with the morphogens, were added directly to the culture medium of the cells in monolayer at concentrations previously shown to be effective in single agent experiments. Cells treated with culture medium or culture medium with the addition of DMSO served as controls. For all experiments, we used the agonists and antagonists in the following concentrations: BMP-2 (20 ng/ml), BMP-7 (30 ng/ml), noggin (100 ng/ml), nodal (30 ng/ml), lefty (100 ng/ml) (all from R&D Systems, Wiesbaden, Germany), and the ALK4/5/7 nodal/activin receptor antagonist SB431542 (30 µM, Sigma-Aldrich).

### Growth assay and chemosensitivity

Melanoma cells (451LU, SKMEL28) or melanocytes were seeded as triplicates in 96-well plates at a density of 1500 cells per well in 150 ml medium (1×10^4^ cells per ml). After 24 h, medium was replaced by medium containing the pharmacological agents (dabrafenib, trametinib) or/and morphogens (BMP-2, BMP-7, noggin, nodal, lefty, SB431542) at the concentrations to be tested. Cells treated with culture medium without or with DMSO served as controls. Assay was started following incubation for 24 h-72 h. Medium was discarded, each well was washed two times with PBS (without Ca^2+^ and Mg^2+^) and 100 ml of a solution containing 100 mg 4-methylumbelliferyl heptanoate per ml PBS was added. Plates were incubated at 37°C for 1 h and measured in a Fluoroskan II (Labsystems, Helsinki, Finland), with an λ_em_ of 355 nm and an λ_ex_ of 460 nm. The intensity of fluorescence indicates the number of viable cells in the wells (Zouboulis et al., 1991).

### Cell-cycle analysis

Cells (451LU, SKMEL28, B16F1, or melanocytes) were pre-treated with morphogens (BMP-2, BMP-7, nodal, noggin, lefty, SB431542) for 24 h. Cells (1×10^6^) were harvested, washed with cold PBS, fixed with 75% ethanol, and incubated at 4°C for at least 1 h. Cells were then centrifuged and washed twice in cold PBS. Intracellular DNA was labelled with propidium iodide solution (propidium iodide 50 µg/ml and RNase 100 µg/ml in PBS) and incubated at 4°C for 30 min in the dark. Cell cycle was analyzed using flow cytometry and FACSDiva software (BD Biosciences). Results were expressed as mean±s.d. values of three independent experiments.

### Colony formation assay

Colony formation assay in agar by SKMEL28 cells was performed as previously described (Sinnberg et al., 2011).

### Boyden chamber invasion assays

Invasion assays of primary melanocytes (control, BMP-2, BMP-7, nodal) using the Boyden chamber were performed as previously described (Sinnberg et al., 2011).

### Organotypic culture of human skin and melanoma cells or melanocytes

Organotypic cultures of human skin and melanoma were generated as described by (Meier et al., 2000). Pre-treated BLM melanoma cell aggregates generated as described by (Drews et al., 1988) were seeded onto human epidermal reconstructs. Untreated BLM cells served as controls. After 16 days of culture, the epidermal skin reconstructs were harvested, fixed with 4% paraformaldehyde for 8 h, dehydrated, and embedded in paraffin. For assessment of invasive tumor growth, 3 µM paraffin sections were generated and stained with Hematoxylin and Eosin (H&E). In a second experiment, untreated, BMP-2- and nodal-pre-treated (24 h) radial growth phase SBCL2 melanoma cells were applied in the same experimental setting. In a third experiment, human melanocytes (untreated, BMP-2, BMP-7, BMP-2+BMP-7+nodal) were applied in the same experimental setting.

### Chick embryo brain metastasis model

Previously generated (Busch et al., 2012; Sinnberg et al., 2018) histological, MIB-1 stained sections of chick embryos 96 h after injection of melanoma cells into the IV^th^ brain ventricle (rhombencephalon) were used to quantify invasion of single melanoma cells (SKMEL28, 451LU, BLM) into the surrounding chick embryo host tissues. All single disseminated melanoma cells that had emigrated from the primary tumor nodule were counted. One representative section per embryo with the largest tumor diameter was analyzed by two independent researchers (T.S. and C.B.).

### BMP-2 ELISA

For the BMP-2 ELISA we used blood samples of the serum bank of the Department of Dermatology, University of Tuebingen, Germany, which was approved by the local ethics committee. No samples were excluded. Plasma BMP-2 levels were determined by using the BMP-2 Quantikine ELISA kit (R&D Systems) according to the manufacturer's instructions.

### BMP-2 real time PCR analysis

For RNA isolation SKMEL28, 451LU and BLM melanoma cell aggregates were used. Total RNA was isolated by using RNeasy Mini Kit (Qiagen) according to the manufacturer’s instructions, including DNase I treatment to remove contaminating genomic DNA (Invitrogen). Purified RNA samples were used for reverse transcription (RT) reaction containing Oligo d(pT)18 mRNA primers (New England Biolabs, Frankfurt, Germany) and Superscript II reverse transcriptase enzyme (Invitrogen) according to the supplier's protocol. The human TGFbeta BMP signaling pathway RT2 Profiler PCR array (Qiagen) comprising a total of 84 genes was performed according to the manufacturer's instructions. For this project, only the data for BMP-2 (CT-values) were included.

### Databases

The following public databases were used to retrieve RNA-expression data in melanoma cells and melanocytes: Zurich biobank database (http://tcgabrowser.ethz.ch:3839/MCE/), phenotype-specific gene expression database (http://www.jurmo.ch/hopp/hopp_mpse.php).

### Statistical analyses

Statistical analyses were performed with a two-tailed unpaired *t*-test or one-way ANOVA for groups more than two. In case of multiple comparisons, Tukey's test was applied. *P*-values <0.05 were considered statistically significant.
